# Duodenal gastrointestinal stromal tumors: clinicopathological characteristics, surgery, and long-term outcome

**DOI:** 10.1186/s12893-015-0084-3

**Published:** 2015-08-15

**Authors:** Chaoyong Shen, Haining Chen, Yuan Yin, Jiaju Chen, Luyin Han, Bo Zhang, Zhixin Chen, Jiaping Chen

**Affiliations:** Department of Gastrointestinal Surgery, West China Hospital, Sichuan University, Chengdu, 610041 Sichuan China; Intensive Care Unit, West China Hospital, Sichuan University, Chengdu, 610041 China

**Keywords:** Duodenum, Gastrointestinal stromal tumor, Clinicopathological Surgery, Prognosis

## Abstract

**Background:**

Duodenal gastrointestinal stromal tumors (DGIST) are rare, and data on their management is limited. We here report the clinicopathological characteristics, different surgical treatments, and long-term prognosis of DGIST.

**Methods:**

Data of 74 consecutive patients with DGIST in a single institution from June 2000 to June 2014 were retrospectively analyzed. The overall survival (OS) and recurrence/metastasis-free survival rates of 74 cases were calculated using Kaplan–Meier method.

**Results:**

Out of 74 cases, 42 cases were female (56.76 %) and 32 cases (43.24 %) were male. Approximately 22.97, 47.30, 16.22, and 13.51 % of the tumors originated in the first to fourth portion of the duodenum, respectively, with a tumor size of 5.08 ± 2.90 cm. Patients presented with gastrointestinal bleeding (*n* = 37, 50.00 %), abdominal pain (*n* = 25, 33.78 %), mass (*n* = 5, 6.76 %), and others (*n* = 7, 9.76 %). A total of 18 patients (24.3 %) underwent wedge resection (WR); 39 patients (52.7 %) underwent segmental resection (SR); and 17 cases (23 %) underwent pancreaticoduodenectomy (PD). The median follow-up was 56 months (1–159 months); 19 patients (25.68 %) experienced tumor recurrence or metastasis, and 14 cases (18.92 %) died. The 1-, 3-, and 5-year recurrence/metastasis-free survival rates were 93.9, 73.7, and 69 %, respectively. The 1-, 3- and 5-year OS were 100, 92.5, and 86 %, respectively. The recurrence/metastasis-free survival rate in the PD group within 5 years was lower than that in the WR group (*P* = 0.047), but was not different from that in the SR group (*P* = 0.060). No statistically significant difference was found among the three operation types (*P* = 0.294).

**Conclusions:**

DGIST patients have favorable prognosis after complete tumor removal, and surgical procedures should be determined by the DGIST tumor location and size.

## Background

Mazur and Clark re-evaluated the histogenesis of gastrointestinal stromal tumors (GIST) in 1983, and following research has confirmed that GIST are the most common mesenchymal tumors [1–4]. Although GIST can originate within the entire gastrointestinal tract, the most common location is the stomach (approximately 60 %), followed by the small intestine (about 20–30 %), and rarely in the duodenum (5 %) [5, 6]. Duodenal gastrointestinal stromal tumor (DGIST) accounted for 10–30 % of all malignant tumors of the duodenum, with a global incidence rate of approximately 10/10^6^–20/10^6^. Nonspecific abdominal pain and gastrointestinal hemorrhage are the most frequent symptoms in GIST patients, and several emergency patients have been admitted to a hospital because of this disease [2, 7].

Primary GIST is categorized into very low, low, intermediate, and high risk based on a previous study of Fletcher [8]. However, subsequent studies have shown that GIST have different clinical, histological, and immunohistochemical features due to different tumor locations; this difference is also one of the independent risk factors for tumor recurrence [9, 10]. To date, surgery with histologically negative margins is mainstream treatment for primary resectable GIST. However, surgical operations for DGIST are often difficult because of anatomical and physiological specificities (the proximity of the head of pancreas, common bile duct, ampullary part, kidney, and mesenteric vessels). There is no consensus on the optimal operation procedures for DGIST at present [11, 12]. Operations vary from a mini-invasive approach to a pancreaticoduodenectomy, which are mainly determined by tumor location and size. Pancreaticoduodenectomy (PD) is considered a safe operation with low mortality; however, PD is highly complicated, and serious immediate and long-term complications occur to some patients [13]. Theoretically, wedge resection (WR) and segmental resection (SR) are simple and feasible. And the main concern on WR or SR is increased risk of tumor recurrence because of incomplete resection [7].

Currently, numerous studies involving DGIST have been published, but most of these studies have small samples or are retrospective case series [6, 14–18]. In the present study, we aimed to evaluate clinical and pathological characteristics, operation curative effects, and long-term prognosis of DGIST patients from a single medical institution.

## Methods

### Patient selection

Medical records of DGIST patients admitted in the Department of General Surgery of West China Hospital of Sichuan University from June 2000 to June 2014 were retrospectively analyzed. Inclusion criteria are as follows: (1)Patients who underwent laparotomy; (2)Patients with DGIST, as proven by pathological, immunohistochemical, and gene mutation detection examinations (spindle cells are observed under microscope; and CD117+ was analyzed through immunohistochemistry or detected through *KIT/PDGFRA* gene mutation, confirmed by senior pathologists); (3)The tumor was located in the duodenum, as confirmed by preoperative abdominal CT scan, ultrasound endoscopy, upper gastrointestinal barium swallow radioscopy, and operation; (4)Patients with GIST synchronous with other malignancies were excluded in this study. A total of 74 DGIST patients were included and examined in this study. The Institutional Review Board and Ethics Committee of the West China Hospital of Sichuan University informed that an ethical review was not needed for this retrospective study.

### Surgery and medication treatment

All patients underwent laparotomy with general anesthesia, and surgical procedures were performed according to intraoperative exploratory results. Surgical procedures were considered to achieve R0 resection as much as possible. Frozen slices of incisal margin and surgical specimen were routinely collected during the surgery. Surgeries included WR (without duodenal transection or anastomosis, local resection with pure closure), SR (duodenal transaction with reconstruction by Roux-en-Y duodenojejunostomy, end-to-end duodenoduodenostomy, or gastrojejunostomy), and PD (operation with pancreaticogastrostomy or pancreatojejunostomy). WR with primary closure was mainly performed for small or abluminal lesions. Tumor risk categories were evaluated according to the modified National Institutes of Health (NIH) risk classification [19]. The patients with intermediate- and high-risk were recommended to take imatinib mesylate (IM) as adjuvant therapy. The recommended IM dosage was 400 mg/d. One patient who received preoperative IM therapy underwent endoscopic ultrasonography-guided fine needle aspiration to confirm the diagnosis, according to the National Comprehensive Cancer Network guideline [20]. All patients signed informed consents and voluntarily accepted the treatment.

### Data collection and follow-up

Data on clinical symptoms, gender, age, hospital stay, surgical procedures (WR, SR, and PD), operation complications (including postoperative abdominal or wound infection, anastomotic fistula, and gastric emptying, etc.), emergency admission, tumor size (maximal tumor diameter, cm), tumor location (first, second, third, and fourth duodenum portion), NIH risk classification, mitotic count per 50 high power fields (HPF) of the microscope, medication before and after surgery (dosage and duration), tumor recurrence/metastasis time, and postoperative follow-up information were collected. All patients were followed up by office visit, telephone call, or outpatient clinic visit after being discharged from the hospital (once every 2–3 months in the first half of the year and then once every 6–12 months a year later). The censor date of the follow-up was July 2014.

### Statistical analysis

Categorical variables were described in terms of frequency and percentages. The measurement data were expressed as mean ± SD. One-way ANOVA was used to compare the clinicopathological characteristics of the three surgical groups. Chi-square test was used to enumerate the data. Wilcoxon test was used to test rank the data. The recurrence/metastasis-free survival rate was measured from operation to tumor recurrence or metastasis (based on radiological findings or proven by biopsy). Overall survival (OS) time includes the period from surgery to death or until the last follow-up. Survival curves were performed using Kaplan–Meier method and compared using log-rank test. Statistical significance was defined as a two-tailed *P* < 0.05. All data analyses were performed using SPSS version 18.0 statistical software package for Microsoft Windows.

## Results

### Patient characteristics

**Fig. 1:**
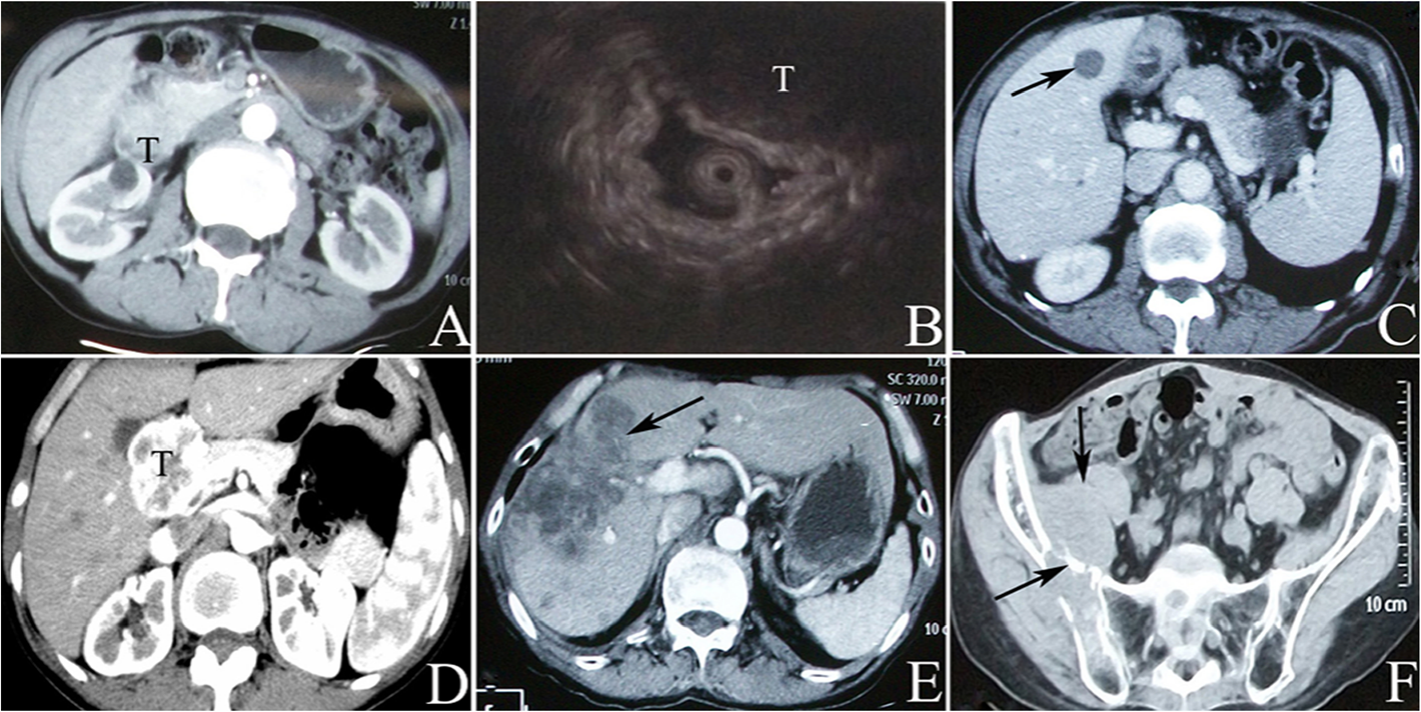
**a**, **b**: The abdominal CT scan and endoscopic ultrasonography images reveal a soft tissue mass with size of 6.5 × 4.5 cm located in the second portion of the duodenum, and with an unclear boundary with head of pancreas, right liver, and kidney. **c** shows a metastasis in the liver at 44 months postoperatively. **d**: A lump with a size of 4.5 × 3.7 cm adjacent to pancreas. **e**, **f**: Liver (mainly in the right liver lobe) and bone (multiple bone destruction of ilium) metastasis occurred 12 years after operation

Table 1 shows the clinicopathological characteristics of the patients. One patient was diagnosed as DGIST preoperatively and had undergone complete tumor resection. However, liver metastasis had occurred at 44 months postoperatively (Fig. 1a–c). Two patients had distant metastasis at the time of diagnosis (a patient with left hepatic metastasis underwent complete resection; no recurrence or metastasis occurred after 25 months of follow-up; another patient had extensive abdominal metastasis and died at 26 months postoperatively). A total of 16 patients received tyrosine kinase inhibitor as adjuvant therapy and were treated for a median time of 28 months (1–52 months). Among them, 1 patient took SUTENT (sunitinib malate, because of intolerance of IM therapy) at 50 mg/d, and 15 patients took imatinib mesylate at 400 mg/day; 6 patients had mild eyelid edema, and 1 patient had mild abnormal liver function. However, these patients did not stop the medication. One patient underwent surgery with complete tumor resection after treatment with IM. However, liver metastasis occurred at 44 months postoperatively. The remaining cases refused to undergo adjuvant therapy mainly due to economic reasons.

**Table 1:** Clinicopathological characteristics and demographic data for patients with duodenal GIST

Variables	No. of patients (*n* = 74)	Percentage (%)
Gender		
Male	32	43.24
Female	42	56.76
Age (years)	54.36 ± 13.13	-
Hospital stay (days)	19.47 ± 10.19	-
Clinical presentation		
Gastrointestinal hemorrhage	37	50.00
Abdominal pain	25	33.78
Palpable mass	5	6.76
others^a^	7	9.46
Tumor location (portion)		
First	17	22.97
Second	35	47.30
Third	12	16.22
Forth	10	13.51
Tumor size (cm)	5.08 ± 2.90	-
No. of mitosis		
≤5/50 HPF	41	55.41
6–10/50 HPF	27	36.49
>10/50 HPF	6	8.10
Modified NIH risk classification		
Low	32	43.24
Intermediate	8	10.81
High	34	45.96
Mutational analysis		
*KIT* exon 11	9	12.16
*KIT* exon 9	3	4.05
NA	62	83.78
Preoperative imatinib therapy	1	1.35
Postoperative adjuvant therapy	16	21.62
Patients with emergency visit	5	6.76
Patients with metastases at diagnosis	2	2.70
Tumor recurrence or metastasis	19	25.68
Death case	14	18.92

GIST, gastrointestinal stromal tumors; ^a^others include jaundice, incidentally found, abdominal distension, *et al*.; HPF, High power field; NIH, National Institutes of Health; NA, not available

### Surgery and postoperative complications

Table 2 summarizes the clinicopathological characteristics of the three types of surgical procedures. Out of the 74 DGIST patients, 18 (24.3 %), 39 (52.7 %), and 17 underwent WR, SR, and PD, respectively. A total of 73 patients obtained R0 resection, and 1 patient in the SR group underwent palliative operation due to extensive abdominal metastasis. Of note, the tumor size in the PD group patients was larger than those in the WR (*P* = 0.005) and SR groups (*P* = 0.011). PDs were mainly chosen for patients with ampullary involvement or close proximity of the tumor to the second segment of the duodenum, whereas those with lesions in the other portion of the duodenum were treated with WR or SR. The hospital stay of patients in the PD group was longer than those in the WR group (*P* = 0.011), but was not significantly different from those in the SR group (*P* = 0.194). No statistically significant difference was noted regarding to gender, age, clinical manifestation, mitotic count (50HPF), and NIH risk classification among the three groups (*P* > 0.05). In addition, 5 patients in the SR and PD groups underwent multivisceral resection respectively; 8 patients underwent cholecystectomy; 1 patient underwent hepatic left lateral lobectomy; and 1 patient had combined right kidney and colon resection. Four patients (23.5 %) experienced postoperative complications in the PD group, such as wound infection (*n* = 2), delayed gastric emptying (*n* = 1), and anastomotic fistula (*n* = 1). In addition, 1 patient from the SR group had intraperitoneal infection, whereas 1 patient in the WR group had intestinal obstruction. The serious complications of reoperation and surgery-related death were not observed among the 3 groups. A total of 3 (16.7 %), 8 (20.5 %), and 4 patients (23.5 %) in the WR, SR, and PD groups, respectively, underwent perioperative blood transfusions.

**Table 2:** Main clinical characteristics and surgical information in three surgical procedures for duodenal GIST

Variables	WR (*n* = 18)	SR (*n* = 39)	PD (*n* = 17)
Gender			
Male (%)	5 (27.8)	19 (48.7)	8 (47.1)
Female (%)	13 (72.2)	20 (51.3)	9 (52.9)
Age (years)	51.50 ± 12.28	49.74 ± 14.60	50.59 ± 13.13
Hospital stay (days)	15.05 ± 2.71	19.36 ± 9.00	22.59 ± 10.88
Clinical presentation			
Gastrointestinal hemorrhage (%)	9 (50.0)	21 (53.8)	9 (52.9)
Abdominal pain (%)	6 (33.3)	9 (23.1)	4 (23.5)
Mass (%)	1 (5.6)	4 (10.3)	3 (17.6)
Others^a^ (%)	2 (11.1)	5 (12.8)	0 (0.0)
Tumor location (portion)			
First (%)	2 (11.1)	14 (35.9)	1 (5.9)
Second (%)	11 (61.1)	11 (28.2)	13 (76.5)
Third (%)	3 (16.7)	7 (17.9)	2 (11.8)
Forth (%)	2 (11.1)	7 (17.9)	1 (5.9)
Tumor size (cm)	4.17 ± 2.83	4.74 ± 2.11	6.84 ± 3.82
No. of mitosis			
≤5/50 HPF (%)	11 (61.1)	23 (59.0)	7 (41.2)
6–10/50 HPF (%)	5 (27.8)	13 (33.3)	9 (52.9)
>10/50 HPF (%)	2 (11.1)	3 (7.7)	1 (5.9)
Modified NIH risk classification			
Low (%)	9 (50.0)	17 (43.6)	6 (35.3)
Intermediate (%)	2 (11.1)	6 (15.4)	0 (0.0)
High (%)	7 (38.9)	16 (41.0)	11 (64.7)
Mutational analysis			
*KIT* exon 11 (%)	0 (0.0)	6 (15.4)	3 (17.6)
*KIT* exon 9 (%)	2 (11.1)	1 (2.6)	0 (0.0)
NA (%)	16 (88.9)	32 (82.1)	14 (82.4)
Margins status			
R0 (%)	18 (100.0)	38 (97.4)	17 (100.0)
R1 (%)	0 (0.0)	1 (2.6)	0 (0.0)
Postoperative complication			
Wound infection (%)	0 (0.0)	0 (0.0)	2 (11.8)
Intra-abdominal infection (%)	0 (0.0)	1 (2.6)	0 (0.0)
Delayed gastric emptying (%)	0 (0.0)	0 (0.0)	1 (5.9)
Intestinal obstruction (%)	1 (5.6)	0 (0.0)	0 (0.0)
Death related surgery (%)	0 (0.0)	0 (0.0)	0 (0.0)
Anastomotic fistula (%)	0 (0.0)	0 (0.0)	1 (5.9)
Multivisceral resection (%)	0 (0.0)	5 (12.8)	5 (29.4)
Perioperative blood transfusion (%)	3 (16.7)	8 (20.5)	4 (23.5)
Tumor recurrence or metastasis (%)	1 (5.6)	11 (28.2)	7 (41.2)

GIST, gastrointestinal stromal tumors; WR, wedge resection; SR, segmental resection (SR); PD, pancreaticoduodenectomy; ^a^others include jaundice, incidentally found, abdominal distension, *et al*.; HPF, High power field; NIH, National Institutes of Health; NA, not available

### Overall and recurrence/metastasis-free survival

**Fig. 2:**
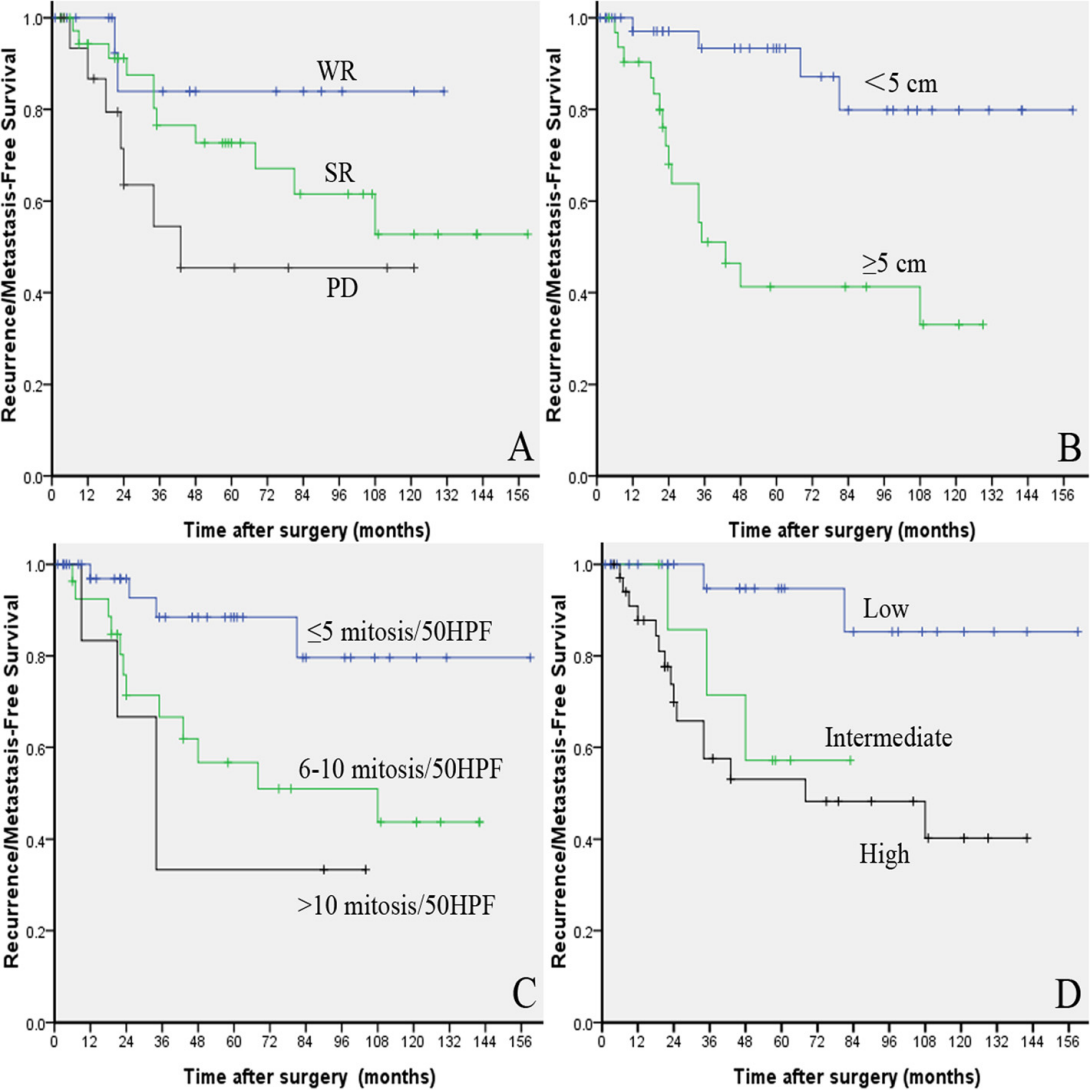
Recurrence/metastasis-free survival of 74 duodenal GIST patients. **a**: stratified by surgical procedures (WR, SR vs. PD). **b**: stratified by tumor size (<5 cm vs. ≥5 cm). **c**: stratified by mitotic count (≤5, 6–10 vs. > 10). **d**: stratified by NIH risk classification (low, intermediate vs. high)

With a median follow-up of 56 months (1–159 months), 7 cases were lost to follow-up. A total of 19 patients (25.68 %) had tumor recurrence or metastasis and 1 patient experienced liver and multiple bone metastases (Fig. 1d–f). Fourteen patients (18.92 %) died. The 1-, 3-, and 5-year recurrence/metastasis-free survival rates were 93.9, 73.7, and 69 %, respectively. The 1-, 3- and 5-year OS were 100, 92.5, and 86 %, respectively. The recurrence/metastasis-free survival of the PD group within 5 years was lower than that of the WR group (*P* = 0.047) but was not significantly different compared with that of the SR group (*P* = 0.060, as shown in Fig. 2). Moreover, the median recurrence/metastasis-free survival in the PD group (22 months) was shorter than that in the SR group (35 months). However, this difference was not statistically significant (*P* = 0.064). Notably, the OS among the three surgical procedures was not statistically significant (*P* = 0.294). The 5-year recurrence/metastasis-free survival rate of patients with tumor size of <5 cm was higher than those with tumor size of ≥5 cm (*P* < 0.001). The 5-year recurrence/metastasis-free survival rate of patients with mitosis count of ≤5, 6–10, and >10 were 88.5 %, 56.7 % (*P* = 0.012), and 33.3 % (*P* = 0.002 for mitotic count ≤ 5, *P* = 0.346 for mitotic count 6–10), respectively. Patients with low-, intermediate-, and high-risk, the 5-year recurrence/metastasis-free survival rates were 94.7 %, 57.1 % (*P* = 0.025), and 53.1 % (*P* = 0.001 for low risk and *P* = 0.364 for intermediate risk), respectively. Patients with intermediate- or high-risk who took IM adjuvant therapy revealed a trend of higher recurrence/metastasis-free survival than that of patients without taking IM (*P* = 0.326).

## Discussion

GIST has been misdiagnosed as smooth muscle or neurogenic tumor for decades because of the insufficient understanding of the GIST concept. However, GIST has been confirmed as an independently oriented differentiated mesenchymal tumor of the digestive tract along with the rapid development of immunohistochemical and molecular biological technologies. To date, most preliminary studies on DGIST are case reports or small sample studies, and only a limited number of studies have reported the operation and long-term prognosis of DGIST [14, 21, 22]. In the present study, the clinicopathological features and long-term prognosis of 74 DGIST patients from a single institution were retrospectively analyzed. This research is important because aside from summarizing the disease’s clinical presentation, the long-term prognosis of the different surgical procedures, and OS and recurrence/metastasis-free survival rate were explored as well.

The clinical presentations of DGIST were non-specific, which were related with the tumor size, growth location, and ulcer in the mucous layer. Alimentary tract hemorrhage is the main clinical manifestation of DGIST, as previously reported in literature [23]. DGIST is mainly located in the duodenum muscle layer and may move inward to the submucosa and lamina propria, leading to mucosal ulceration and hemorrhage [24]. Similar to two recent reports [25, 26], the tumors were mainly located in the second portion of the duodenum (47.30 %), followed by the first portion (22.97 %). Furthermore, we have found that the PD ratio in the second portion of the duodenum was higher than that in other portions (76.5 %). The tumor around this site was often located in the inner or posteromedial side of the second portion of DGIST, and the ampulla of Vater and pancreatic head were involved. Our data showed that the low mitotic count proportion (≤5/50 HPF) in DGIST patients was higher (55.41 %) than patients with GIST in the stomach and small intestine; this result is similar with previous reports [12, 23, 26]. The tumor (4–5 cm) of DGIST patients at diagnosis was smaller than tumors in the stomach or other sites [23, 27]. The tumor size was 5.08 ± 2.90 cm in this cohort, which was agreement with their reports.

Currently, surgery is the only potential curative treatment of GIST if R0 is performed [7]. But surgical resection is difficult, and digestive tract reconstruction is not easy for DGIST. Generally, the choice of surgical approach depends on the tumor site, size, and invasion degree into adjacent organs. In this cohort, a total of 10 patients underwent combined organ resection, and all of them obtained R0 resection. Patients who had PD were more likely to experience a higher risk of postoperative complications. The present study also confirmed this finding (PD, SR, WR: 23.5, 2.6, 5.6 %). Patients with larger tumors or lesions located in the second portion of the duodenum, the probability of undergoing PD increased. Notably, no surgery-related death was noted in the three surgical procedures. Thus, we assumed that these three procedures for DGIST were safe and reliable. However, there were still disagreements over the optimal surgical procedures for DGIST [12, 23, 24]. Furthermore, WR or SR can also be safely performed by means of laparoscopic and robotic approaches for DGIST [28, 29]. GIST seldom occurs in lymph node metastasis or peritoneal metastasis; therefore, extensive lymphadenectomy was unnecessary. In this study, only one patient had distant multiple bone metastases.

In the current series, the 5-year OS and recurrence/metastasis-free survival rates were 86 and 69 %, respectively, which were similar to the results of other reports [23, 25]. By contrast, the 5-year disease-free survival rate of small intestine GIST in other parts was lower (about 40 %) compared with DGIST patients [30]. A favorable prognosis of DGIST patients may be related with the early clinical symptoms and small diameter tumor. We found that the type of surgical procedure can affect outcomes of DGIST patients to a certain extent, and this finding is consistent with the results of Colombo et al. [25]. However, Johnston et al. showed that the DGIST recurrence depends on tumor biology, rather than the operation type or microscopic margins [26]. This phenomenon could be attributed to the fact that the tumor size in the PD group was often larger than that of the other two groups in this study. It is well known that tumor size was one of most important prognostic indicator for GIST.

Nowadays, IM played a key role in the management of GIST when used as adjuvant therapy. The intermediate-risk subgroup of GIST Patients should take IM at least 1 year, while patients with high risk should last for at least 3 years postoperatively [31, 32]. In this study, a total of 16 patients with intermediate- or high-risk underwent adjuvant therapy by using tyrosine kinase inhibitors with a median time of 28 months (1–52 months). We have observed that the recurrence/metastasis-free survival rate of patients with intermediate- or high-risk who underwent postoperative adjuvant therapy was higher than that of patients who did not undergo adjuvant therapy. But no significant difference was noted, thus preventing us from drawing any conclusions. This finding could be attributed to the fact that small number of patients underwent adjuvant therapy and short medication duration. The possibility of complete resection can be evaluated by auxiliary examination preoperatively. Preoperative IM treatment could reduce the proportion of multi-organ resection, downstage giant tumors and increase the opportunity of R0 resection [33, 34]. In this study, one patient underwent preoperative IM treatment and obtained complete resection, thus avoiding combined organ resection and PD.

## Conclusion

In sum, patients with DGIST have a favorable prognosis after complete resection. The 5-year recurrence/metastasis-free survival and OS rates of DGIST were 69 and 86 %, respectively. The treatment of choice for DGIST should be selected according to the DGIST tumor site and size. This study showed that the PD group has a higher complication rate than the WD group, and patients of the former group experienced prolonged hospitalization. In addition, the recurrence/metastasis-free survival rate within 5 years was lower than that of the WR group, but had no significant difference with the SR group.

## References

[1] Mazur MT, Clark HB (1983). Gastric stromal tumors. Reappraisal of histogenesis. Am J Surg Pathol.

[2] Liegl-Atzwanger B, Fletcher JA, Fletcher CD (2010). Gastrointestinal stromal tumors. Virchows Arch.

[3] Corless CL, Barnett CM, Heinrich MC (2011). Gastrointestinal stromal tumours: origin and molecular oncology. Nat Rev Cancer.

[4] Kiśluk J, Gryko M, Guzińska-Ustymowicz K (2013). Immunohistochemical diagnosis of gastrointestinal stromal tumors-an analysis of 80 cases from 2004 to 2010. Adv Clin Exp Med.

[5] Grotz TE, Donohue JH (2011). Surveillance strategies for gastrointestinal stromal tumors. J Surg Oncol.

[6] Buchs NC, Bucher P, Gervaz P, Ostermann S, Pugin F, Morel P (2010). Segmental duodenectomy for gastrointestinal stromal tumor of the duodenum. World J Gastroenterol.

[7] Gervaz P, Huber O, Morel P (2009). Surgical management of gastrointestinal stromal tumours. Br J Surg.

[8] Fletcher CD, Berman JJ, Corless C, Gorstein F, Lasota J, Longley BJ, et al. Diagnosis of gastrointestinal stromal tumors: A consensus approach. Hum Pathol. 2002;33:459–65. 10.1053/hupa.2002.123545 12094370

[9] Wasag B, Debiec-Rychter M, Pauwels P, Stul M, Vranckx H, Oosterom AV (2004). Differential expression of KIT/PDGFRA mutant isoforms in epithelioid and mixed variants of gastrointestinal stromal tumors depends predominantly on the tumor site. Mod Pathol.

[10] Miettinen M, Lasota J (2006). Gastrointestinal stromal tumors: pathology and prognosis at different sites. Semin Diagn Pathol.

[11] Zhong Y, Deng M, Liu B, Chen C, Li M, Xu R. Primary gastrointestinal stromal tumors: Current advances in diagnostic biomarkers, prognostic factors and management of its duodenal location. Intractable Rare Dis Res. 2013;2:11-7. 10.5582/irdr.2013.v2.1.11 PMC4204577 25343095

[12] Machado NO, Chopra PJ, Al-Haddabi IH, Al-Qadhi H (2011). Large duodenal gastrointestinal stromal tumor presenting with acute bleeding managed by a whipple resection. A review of surgical options and the prognostic indicators of outcome. JOP.

[13] Cameron JL, Riall TS, Coleman J, Belcher KA (2006). One thousand consecutive pancreaticoduodenectomies. Ann Surg.

[14] Bourgouin S, Hornez E, Guiramand J, Barbier L, Delpero JR, Le Treut YP (2013). Duodenal gastrointestinal stromal tumors (GISTS): arguments for conservative surgery. J Gastrointest Surg.

[15] Liang X, Yu H, Zhu LH, Wang XF, Cai XJ (2013). Gastrointestinal stromal tumors of the duodenum: surgical management and survival results. World J Gastroenterol.

[16] Cassier PA, Blay JY (2011). Gastrointestinal stromal tumors of the stomach and duodenum. Curr Opin Gastroenterol.

[17] Zhou B, Zhang M, Wu J, Yan S, Zhou J, Zheng S (2013). Pancreaticoduodenectomy versus local resection in the treatment of gastrointestinal stromal tumors of the duodenum. World J Surg Oncol.

[18] Hoeppner J, Kulemann B, Marjanovic G, Bronsert P, Hopt UT (2013). Limited resection for duodenal gastrointestinal stromal tumors: Surgical management and clinical outcome. World J Gastrointest Surg.

[19] Joensuu H (2008). Risk stratification of patients diagnosed with gastrointestinal stromal tumor. Hum Pathol.

[20] Demetri GD, von Mehren M, Antonescu CR, DeMatteo RP, Ganjoo KN, Maki RG, et al. NCCN task force report: update on the management of patients with gastrointestinal stromal tumors. J Nat Compr Canc Netw. 2010;8:S1–S41. 10.6004/jnccn.2010.0116 PMC4103754 20457867

[21] Miki Y, Kurokawa Y, Hirao M, Fujitani K, Iwasa Y, Mano M (2010). Survival analysis of patients with duodenal gastrointestinal stromal tumors. J Clin Gastroenterol.

[22] Sakata K, Nishimura T, Okada T, Nakamura M (2009). Local resection and jejunal patch duodeno-plasty for the duodenal gastrointestinal stromal tumor--a case report. Gan To Kagaku Ryoho.

[23] Chung JC, Chu CW, Cho GS, Shin EJ, Lim CW, Kim HC (2010). Management and outcome of gastrointestinal stromal tumors of the duodenum. J Gastrointest Surg.

[24] Miettinen M, Kopczynski J, Makhlouf HR, Sarlomo-Rikala M, Gyorffy H, Burke A (2003). Gastrointestinal stromal tumors, intramural leiomyomas, and leiomyosarcomas in the duodenum: a clinicopathologic, immunohistochemical, and molecular genetic study of 167 cases. Am J Surg Pathol.

[25] Colombo C, Ronellenfitsch U, Yuxin Z, Rutkowski P, Miceli R, Bylina E (2012). Clinical, pathological and surgical characteristics of duodenal gastrointestinal stromal tumor and their influence on survival: a multi-center study. Ann Surg Oncol.

[26] Johnston FM, Kneuertz PJ, Cameron JL, Sanford D, Fisher S, Turley R (2012). Presentation and Management of Gastrointestinal Stromal Tumors of the Duodenum: A Multi-Institutional Analysis. Ann Surg Oncol.

[27] Yang WL, Yu JR, Wu YJ, Zhu KK, Ding W, Gao Y (2009). Duodenal gastrointestinal stromal tumor: clinical, pathologic, immunohistochemical characteristics, and surgical prognosis. J Surg Oncol.

[28] Kato M, Nakajima K, Nishida T, Yamasaki M, Nishida T, Tsutsui S (2011). Local resection by combined laparoendoscopic surgery for duodenal gastrointestinal stromal tumor. Diagn Ther Endosc.

[29] Downs-Canner S, Van der Vliet WJ, Thoolen SJ, Boone BA, Zureikat AH, Hogg ME, et al. Robotic Surgery for Benign Duodenal Tumors. J Gastrointest Surg. 2015;19:306–12. 10.1007/s11605-014-2668-0 PMC4529990 25348238

[30] Rutkowski P, Nowecki ZI, Michej W, Debiec-Rychter M, Woźniak A, Limon J (2007). Risk criteria and prognostic factors for predicting recurrences after resection of primary gastrointestinal stromal tumor. Ann Surg Oncol.

[31] Sjölund K, Andersson A, Nilsson E, Nilsson O, Ahlman H, Nilsson B (2010). Downsizing treatment with tyrosine kinase inhibitors in patients with advanced gastrointestinal stromal tumors improved resectablity. World J Surg.

[32] Joensuu H, Eriksson M, Sundby Hall K, Hartmann JT, Pink D, Schütte J (2012). One vs three years of adjuvant imatinib for operable gastrointestinal stromal tumor: a randomized trial. JAMA.

[33] Blesius A, Cassier PA, Bertucci F (2011). Neoadjuvant imatinib in patients with locally advanced GIST in the prospective BFR 14 trial. BMC Cancer.

[34] Eisenberg BL, Trent JC (2011). Adjuvant and neoadjuvant imatinib therapy: current role in the management of gastrointestinal stromal tumors. Int J Cancer.

